# Optimization of NO oxidation by H_2_O_2_ thermal decomposition at moderate temperatures

**DOI:** 10.1371/journal.pone.0192324

**Published:** 2018-04-18

**Authors:** Hai-qian Zhao, Zhong-hua Wang, Xing-cun Gao, Cheng-hao Liu, Han-bing Qi

**Affiliations:** Department of Civil Engineering & Architecture, Northeast Petroleum University, Daqing, China; Heidelberg University, UNITED STATES

## Abstract

H_2_O_2_ was adopted to oxidize NO in simulated flue gas at 100–500°C. The effects of the H_2_O_2_ evaporation conditions, gas temperature, initial NO concentration, H_2_O_2_ concentration, and H_2_O_2_:NO molar ratio on the oxidation efficiency of NO were investigated. The reason for the narrow NO oxidation temperature range near 500°C was determined. The NO oxidation products were analyzed. The removal of NO*x* using NaOH solution at a moderate oxidation ratio was studied. It was proven that rapid evaporation of the H_2_O_2_ solution was critical to increase the NO oxidation efficiency and broaden the oxidation temperature range. the NO oxidation efficiency was above 50% at 300–500°C by contacting the outlet of the syringe needle and the stainless-steel gas pipe together to spread H_2_O_2_ solution into a thin film on the surface of the stainless-steel gas pipe, which greatly accelerated the evaporation of H_2_O_2_. The NO oxidation efficiency and the NO oxidation rate increased with increasing initial NO concentration. This method was more effective for the oxidation of NO at high concentrations. H_2_O_2_ solution with a concentration higher than 15% was more efficient in oxidizing NO. High temperatures decreased the influence of the H_2_O_2_ concentration on the NO oxidation efficiency. The oxidation efficiency of NO increased with an increase in the H_2_O_2_:NO molar ratio, but the ratio of H_2_O_2_ to oxidized NO decreased. Over 80% of the NO oxidation product was NO_2_, which indicated that the oxidation ratio of NO did not need to be very high. An 86.7% NO removal efficiency was obtained at an oxidation ratio of only 53.8% when combined with alkali absorption.

## Introduction

SO_2_ and NO*x* are two major pollutants released by coal combustion. Since most NO*x* in coal-fired flue gas is NO, which is difficult to directly absorb with a liquid absorbent,[1] the removal of NO*x* in flue gas is more difficult than that of SO_2_. NO*x* has surpassed SO_2_ as the largest gaseous pollutant emission in recent years in China.[2] NO*x* emissions have reached twice that of SO_2_ in some Chinese cities.[2] Therefore, China revised the emission standards for pollutants from thermal power plants in 2011, in which the NO*x* emission standard was changed to 100 mg/Nm^3^ for most pulverized-coal boilers.[3] It is imperative to develop economical and efficient NO*x* removal technology.

At present, successfully commercialized denitrification technologies include low NO*x* burner technology (LNB), selective catalytic reduction technology (SCR) and selective non-catalytic reduction technology (SNCR). SCR can achieve high NO*x* removal efficiency; however, the initial investment and operational cost are expensive.[4] In addition, SCR has secondary pollutions, such as ammonia leak and waste catalyst. LNB can control the emission of NO*x* from a combustion source without greatly increasing the cost. In recent years, LNB technology has made great progress, and it can control the emission concentration of NO*x* to below 300 mg/Nm^3^ in the flue gas from boilers burning bituminous coal.[5]Therefore, it is a promising NO*x* control technology that should be widely developed.

Wet flue gas desulfurization (WFGD) units are commonly installed in thermal power plants. To realize the simultaneous removal of SO_2_ and NO*x* in present WFGD units based on LNB technology will be an economical way to meet the new NO*x* emission standard. However, most NO*x* in coal-fired flue gas is NO, which is difficult to absorb in SO_2_ scrubbers. Developing low-cost and high-efficiency NO oxidization technology is key to realizing the simultaneous removal of SO_2_ and NO*x* in WFGD units.

H_2_O_2_ is widely used as an oxidizing agent for its strong oxidation ability and environmental friendliness. Since the 1990s, researchers have injected H_2_O_2_ solution into flue gas to oxidize NO and gained many valuable results.[1,6–8] Zamansky and coworkers [6] found that the maximal conversion efficiency of NO can reach 90% at 500°C, when the molar ratio of H_2_O_2_:NO was 1.5. Kasper and coworkers[7] achieved a 97% oxidization efficiency of NO when the molar ratio of H_2_O_2_:NO was 2.6. Collins *et al*. [8] achieved high conversions of NO using molar ratios of H_2_O_2_:NO*x* slightly above 1.0. These studies have two similarities. First, the optimum temperatures were 500°C, and when the temperature deviated from 500°C, the NO oxidation efficiency dropped sharply. Second, NO was proven to be oxidized to NO_2_, HNO_2_, and HNO_3_; however, the proportion of various products was not determined. Thus, all researchers aimed for a high oxidization ratio of NO (the molar ratio of oxidized NO to total amount of NO in the flue gas).

For power plant boilers, it is difficult to find a position where the flue gas temperature is exactly 500°C because the flue gas temperature changes greatly over the course of flow. In addition, even in the same flue section, the flue gas temperature varies greatly. However, flue gas with temperature of 500°C can be found in economizers, but it is difficult to inject H_2_O_2_ solution at this site. The temperature of flue gas from the outlet of an economizer is approximately 400°C or lower. If the temperature range over which H_2_O_2_ efficiently oxidizes NO can be expanded, the possible application of this technology will be greatly increased.

The capacity for H_2_O_2_ to oxidize NO mainly comes from the free radicals generated from the decomposition of H_2_O_2_, such as hydroxyl radicals (·OH) and hydroperoxyl radicals (HO_2_·).[9–11] H_2_O_2_ thermal decomposition is one of the simplest means to generate ·OH. The bond length of HO-OH is 148 pm, and the bond dissociation energy is 213.8 kJ/mol.[12] H_2_O_2_ can be quickly decomposed below 500°C, even if it is not homogeneous. Lin *et al*. [13] studied the decomposition kinetics of H_2_O_2_ at 100–280°C. They found that the decomposition of H_2_O_2_ was a first-order reaction regardless of the temperature and reactor type, and the temperature and the reactor type had a great influence on the H_2_O_2_ decomposition rate. The results also showed that the H_2_O_2_ decomposition rate was fast at 280°C (more than 97% of the H_2_O_2_ decomposed in 50 s in Titanium tubing). Mok *et al*. [14] studied the decomposition characteristics of H_2_O_2_in propulsion system (on the surface of silver screen catalyst bed) at higher pressures. It was shown that the decomposition of H_2_O_2_ changed from a heterogeneous reaction to a homogeneous reaction at the temperature of 427°C. Even if the temperature was below 427°C, H_2_O_2_ can still decompose quickly (k = 10^l3^exp(-48000/RT) s^-1^).[15] Ali-zade[16] investigated the gas-phase oxidation reaction kinetics of pyridine derivatives with H_2_O_2_. The H_2_O_2_ concentration was 20–30%, and the temperature was 250–400°C.The results showed that H_2_O_2_ can decompose to generate free radicals efficiently at this temperature.

According to the homogeneous oxidation mechanism of NO by H_2_O_2_,[1] the NO oxidation product should mainly be NO_2_. Under the effect of thermal energy, hydrogen peroxide decomposes, and ·OH radicals are generated. Then, NO rapidly reacts with ·OH to produce HONO. However, the generated HONO reacts with ·OH, and the reaction rate constant k of this reaction is high (k = 6.24×10^−12^(T/298 K)e^-0.57^ cm^3^/molecule·s).[17] The products are NO_2_ and H_2_O. A reaction between H_2_O_2_ and ·OH (reaction 4) also occurs after reaction 1.[18] HO_2_· generated from reaction 4 can oxidize NO to NO_2_.[19] It can be seen that the final product of the reaction between NO and ·OH or HO_2_· should be NO_2_. Thomas and Vanderschuren[20] studied nitrogen oxide scrubbing with alkaline solutions and found that the total dissolved NO*x* concentration reaches a maximum value for intermediate oxidation ratios (50%-70%). This indicates that if NO can be mainly oxidized to NO_2_, the oxidation ratio does not need to be very high, and 50% may be an ideal oxidation ratio for NO*x* absorption in WFGD units.

$$H_{2}O_{2}\to \cdot OH+\cdot OH$$ (1)

NO+⋅OH→HONO (2)

$$\mathrm{HONO}+\cdot \mathrm{OH}\to {\mathrm{NO}}_{2}{+H}_{2}O$$ (3)

$$H_{2}O_{2}+\cdot \mathrm{OH}\to {\mathrm{HO}}_{2}\cdot {+H}_{2}O$$ (4)

$${\mathrm{NO+HO}}_{2}\cdot \to \cdot {\mathrm{OH}+\mathrm{NO}}_{2}$$ (5)

In this research, the NO oxidation efficiency under different H_2_O_2_ evaporation conditions was studied. On this basis, influences of the gas temperature (100–500°C), initial NO concentration, H_2_O_2_ concentration, and H_2_O_2_:NO molar ratio on the oxidation efficiency of NO were investigated in a drop-tube furnace. The NO oxidation products were analyzed. The removal of NO*x* using sodium hydroxide solution was studied at an oxidation ratio of 53.8%.

## Experimental

**Fig 1:**
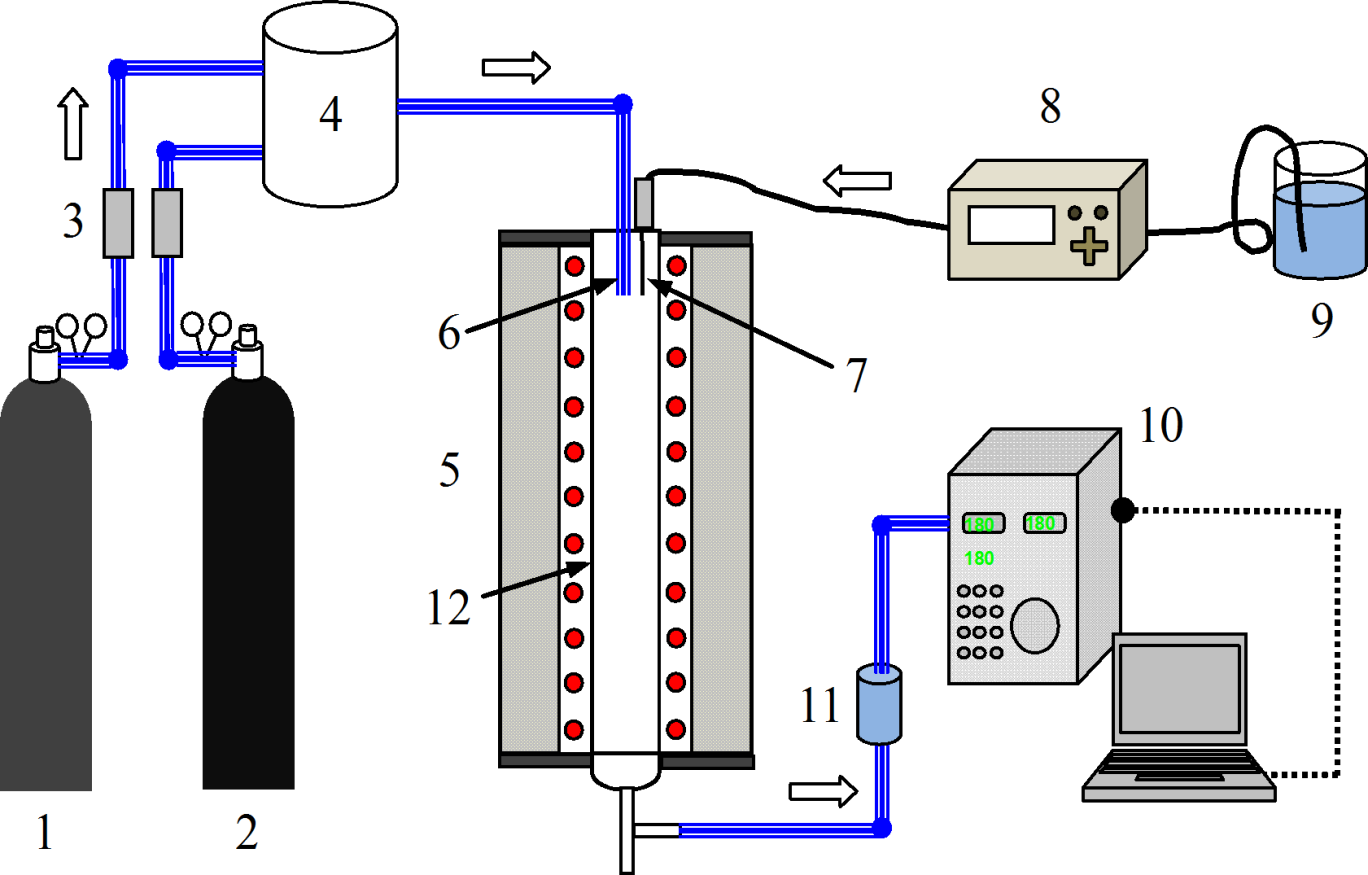
Drop-tube furnace NO oxidization experimental system. 1-NO; 2-N_2_; 3-gas mass flow meter; 4-buffer bottle (D = 100mm. H = 150mm); 5-drop-tube furnace; 6-stainless-steel gas inlet;7-syringe needle;8-micro-membrane pump; 9-H_2_O_2_ solution container; 10-gas analyzer; 11-condenser; 12-quartz oxidation reactor.

NO oxidation by H_2_O_2_ thermal decomposition was performed in a drop-tube furnace NO oxidization experimental system, as shown in Fig 1. The reaction system is composed of a drop-tube furnace, a quartz oxidation reactor, a micro-membrane pump, a H_2_O_2_ solution container, a gas analyzer, a condenser and a gas distribution system. The length of the oxidation reactor was 680 mm, and the inner diameter was 50 mm.

**Fig 2:**
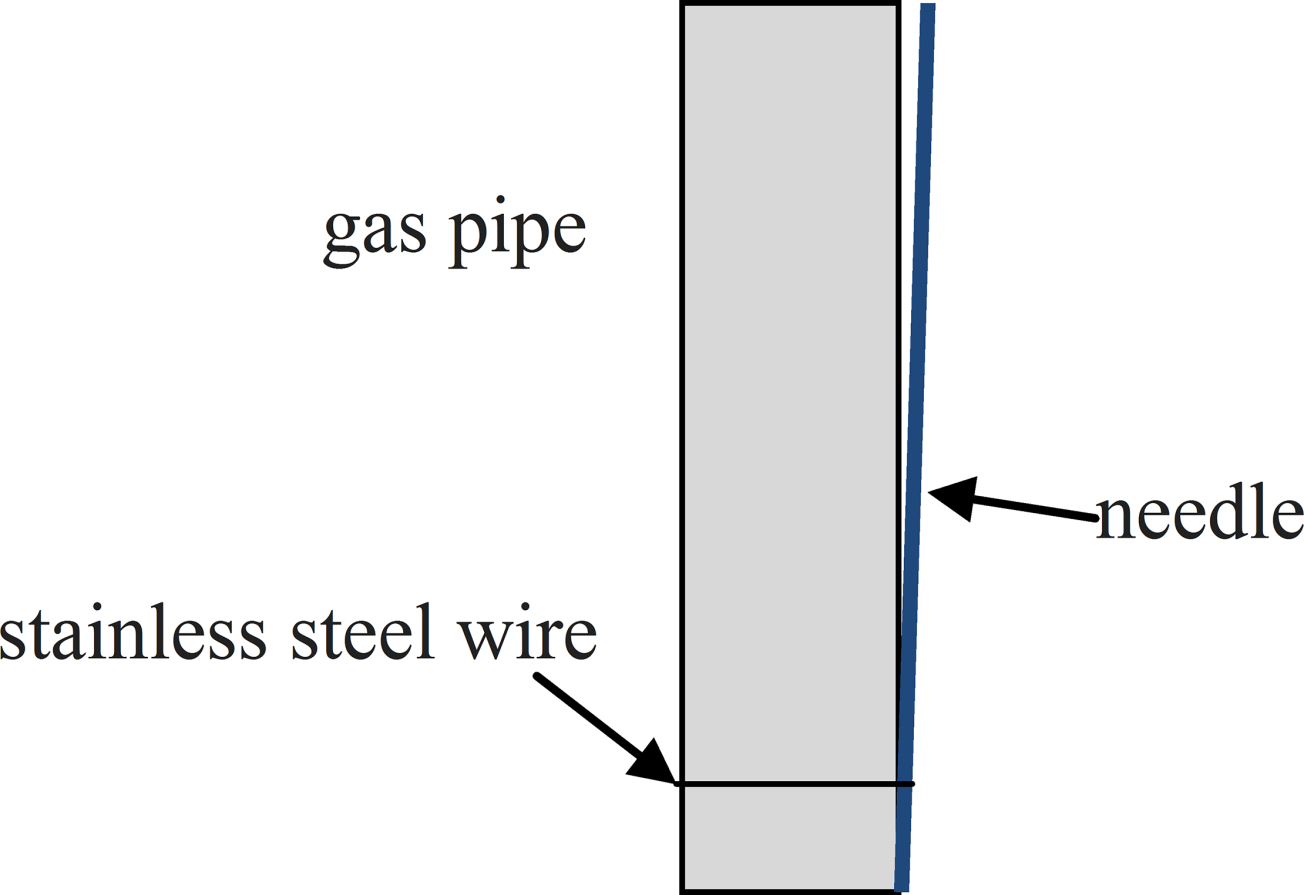
Connection method of the syringe needle and the stainless-steel gas pipe.

The temperature inside the drop-tube furnace was kept at a constant value. N_2_+NO gas with different NO concentrations was provided by an N_2_ gas cylinder and an NO gas cylinder via gas mass flow meters at room temperature (25°C). The total gas flow was 1.5 L/min. N_2_+NO gas was delivered into the reactor through the stainless-steel pipe at the top of the quartz reactor. The pressure inner the reactor was 0.2MPa. H_2_O_2_ solution with a desired concentration was prepared and pumped into the reactor through a long syringe needle using a micro-syringe pump (LSP01-1A, Longer, China). In most experiments, the initial concentrations of H_2_O_2_ were 30%, and the H_2_O_2_:NO molar ratio was 10:1. The injected H_2_O_2_ solution vaporized and decomposed immediately in the oxidation reactor. Then, NO was oxidized by the free radicals formed from the decomposition of H_2_O_2_. To speed the evaporation rate of H_2_O_2_ droplets, the outlet of the syringe needle and the stainless-steel gas pipe were connected (Fig 2). This set-up rapidly converted the H_2_O_2_ droplet into a thin film on the surface of the stainless-steel gas pipe, which prevented H_2_O_2_ droplets from dripping directly to the bottom of the furnace without evaporation.

The pH value of H_2_O_2_ solution bought from Aladdin Industrial Corporation was adjusted to be about 5, and certain stabilizer was added. Under such conditions, H_2_O_2_ was stable at room temperature without catalysts. The H_2_O_2_ solution container was made of glass and the syringe was made of plastic. The connecting tubes in these experiments were mainly silicone tubes. So, the decomposition of H_2_O_2_in the tubes and in the syringe was negligible.

Before the experiment, the NO and N_2_ valves were opened, and N_2_+NO gas flowed into the oxidation reactor. The NO concentration was recorded by the gas analyzer (VARIO PLUS, MRU, Germany). The gas analyzer worked based on the electrochemical principle. NO gas was measured with 3-electrodes sensors.The gas valve opening was adjusted so that the total gas volume was 1.5 L/min and the initial concentration of NO reached 300 mg/m^3^. When the NO concentration was stable, the initial NO gas concentration was recorded. Then, H_2_O_2_ solution was injected into the reactor. H_2_O_2_ was heated and decomposed, and NO was oxidized. The outlet concentration of NO was monitored by the gas analyzer. The oxidation efficiency of NO could be calculated by the NO concentrations.

$$\eta =\frac{C_{0}-C_{1}}{C_{0}}\times 100\%$$

where *η* represents the oxidation efficiency of NO, %; *C*_0_ represents the initial concentration of NO, mg/m^3^; and *C*_1_ represents the average concentration of NO in the reaction process, mg/m^3^.

## Results

### Effect of evaporation of H_2_O_2_solution on the gas temperature

**Fig 3:**
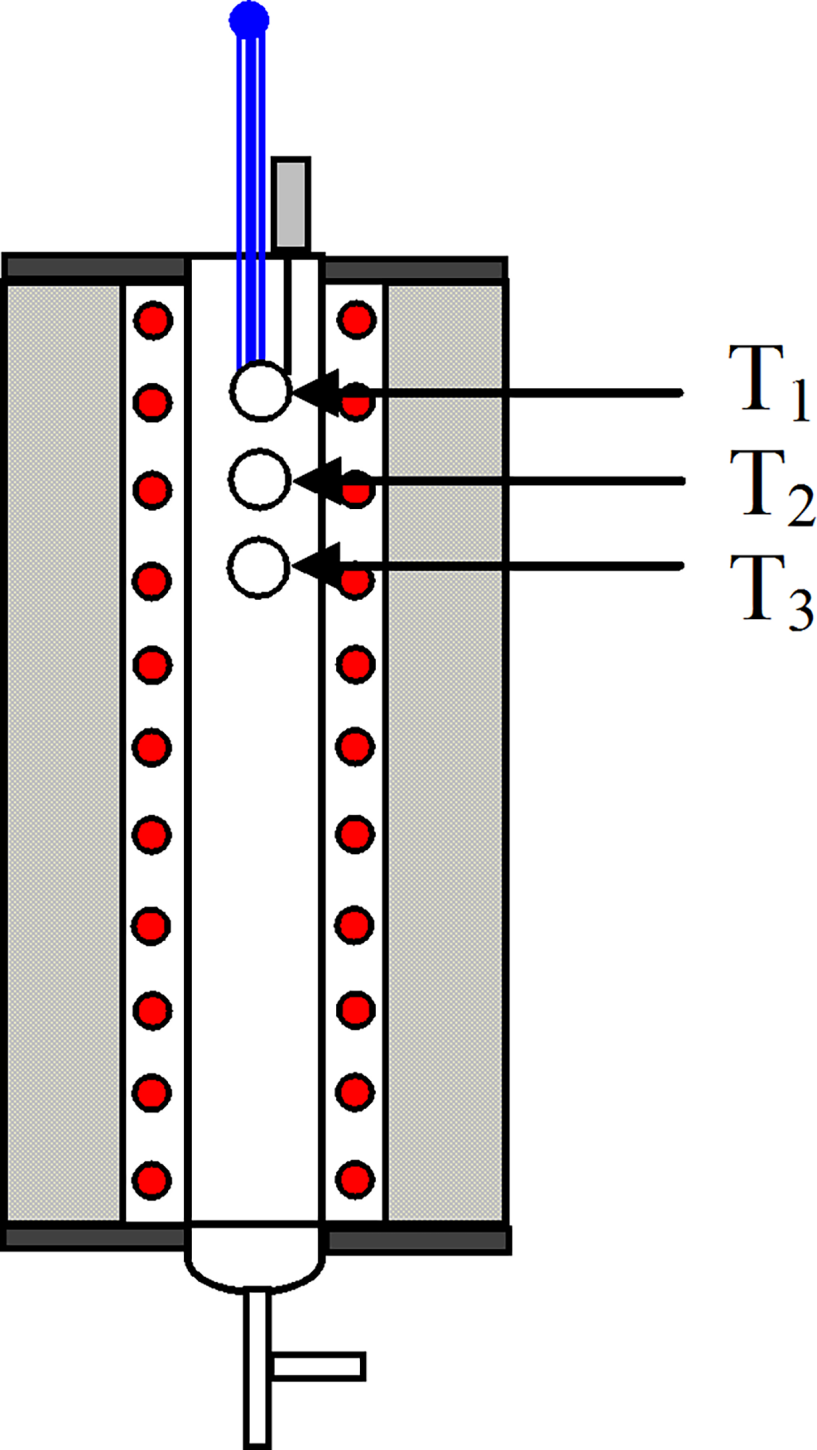
Temperature measuring point positions in the reactor.

A drop-tube furnace was used to maintain the reactor temperature at a constant value in these experiments, but the H_2_O_2_ solution was quickly evaporated, and it absorbed much of the latent heat of vaporization in a short time. This may have an effect on the gas temperature. The influence of H_2_O_2_ solution evaporation on the vertical temperature field in the reactor was studied. The temperatures of three different heights in the reactor were measured by a thermocouple. The positions of the measuring points are shown in Fig 3. The height of measuring point T_1_ was the same as that of the stainless-steel gas inlet. The measuring point T_2_ was 50 mm lower than the gas inlet. The height of the measuring point T_3_ was 50 mm lower than that of T_2_. The temperature of the reactor was 400°C. The flow of H_2_O_2_ solution was 0.08 mL/min, which was the maximum flow in this study. To ensure that the H_2_O_2_ solution was fully evaporated, the H_2_O_2_ syringe needle was connected to the stainless-steel gas inlet. The temperature test results at different measuring points are shown in Table 1.

**Table 1:** Temperature test results at different measuring points.

Temperature measuring point	Distance between the gas inlet(mm)	Temperature before injecting H_2_O_2_ solution(°C)	Temperature after injecting H_2_O_2_ solution(°C)
T_1_	0	398.6	382.5
T_2_	50	399.7	399.2
T_3_	100	400.2	400.5

The results showed that the temperatures at the three measuring points were approximately 400 °C before the H_2_O_2_ solution was injected. As the measuring point T_1_ was located at the end of the furnace, the temperature was slightly lower than 400 °C (398.6 °C). This indicated that the internal temperature in the reactor was uniform. When the H_2_O_2_ solution was injected, the temperature at the T_1_ measuring point decreased significantly (382.5 °C). This was because measuring point T_1_ was located near the inlet of the H_2_O_2_ solution and the gas, where the H_2_O_2_ solution was injected and evaporated rapidly. Evaporation of the H_2_O_2_ solution quickly absorbed a large amount of heat, resulting in a large decrease in the temperature near the T_1_ measuring point. However, the temperature away from T_1_ quickly reached 400 °C. The temperature at T_2_ and T_3_ was close to 400 °C. It can be seen that the evaporation of H_2_O_2_ solution only affected the gas temperature near the gas inlet, and the temperature of the whole reactor was not affected.

Since the needle diameter was too small, it was difficult to measure its surface temperature. However, the height of the measuring point T_1_ was the same as that of the needle outlet, so the needle outlet temperature should be close to 382.5 °C after H_2_O_2_ solution was injected.

### Effect of H_2_O_2_ solution evaporation

**Fig 4:**
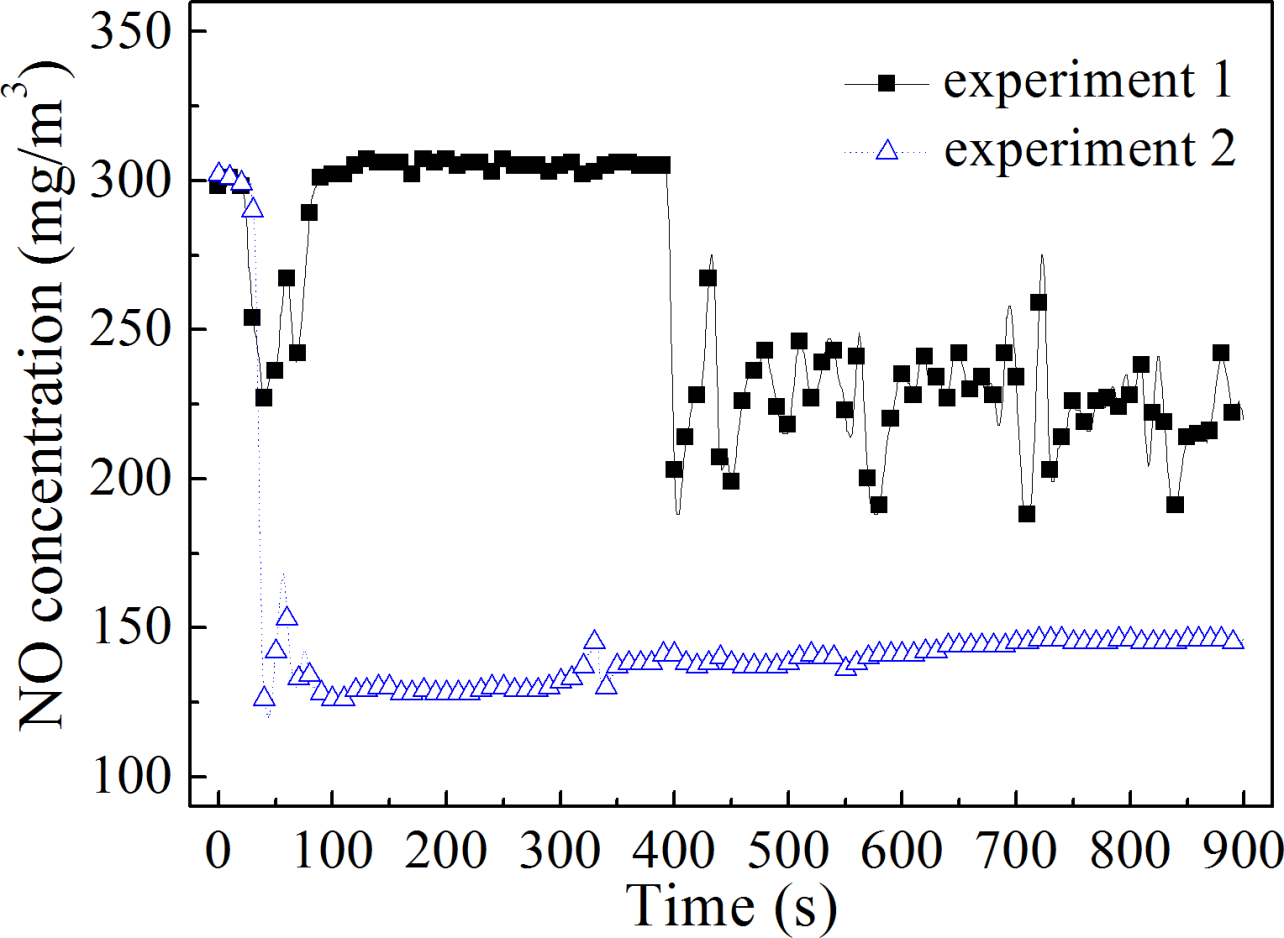
Influence of H_2_O_2_ evaporation on NO oxidation. The temperature in the reactor was 400°C.

The evaporation of H_2_O_2_ solution had an important effect on the thermal decomposition of H_2_O_2_. If the H_2_O_2_ solution did not evaporate quickly, the H_2_O_2_ solution would form large droplets at the needle of the syringe. When the droplets grow large enough, they would drip rapidly, resulting in a very short residence time of H_2_O_2_ in the oxidation reactor. In this case, H_2_O_2_ could not be decomposed effectively. Only when the H_2_O_2_ solution was fully evaporated could H_2_O_2_ decompose and oxidize NO gas quickly. Therefore, NO concentrations were compared under different evaporation conditions. In experiment 1, the gas inlet and needle were not in direct contact, and H_2_O_2_ solution droplets dripped quickly through the reactor. In experiment 2, the H_2_O_2_ syringe needle was connected to the stainless-steel gas inlet, which promoted the rapid decomposition of H_2_O_2_. Fig 4 shows the effect of H_2_O_2_ solution evaporation on NO oxidization.

In experiment 1, the H_2_O_2_ solution did not evaporate rapidly. Most of the H_2_O_2_ was discharged from the bottom of the reactor in liquid form. Although the NO concentration decreased after the injection of H_2_O_2_, the NO concentration in the whole process did not remain stable, which reduced the average oxidation efficiency of NO. When the droplets evaporate well (in experiment 2), the concentration of NO gas decreased sharply and remained constant after the H_2_O_2_ solution was injected into the reactor. This indicated that the thermal decomposition of H_2_O_2_ was stable and that the decomposition products could oxidize NO efficiently. The results proved that sufficient evaporation of H_2_O_2_ solution was a prerequisite for high oxidation efficiency of NO. In the latter experiments, the syringe needle was connected to the stainless-steel gas inlet tube to ensure that the H_2_O_2_ solution was fully evaporated.

Collins *et al*. [8] injected a H_2_O_2_ solution into hot flue gas to control NO*x* emission. They found that the position of the injector and the type of atomization were very important to the efficient utilization of H_2_O_2_. This was most likely due to the position of the injector and the type of atomization affecting H_2_O_2_ evaporation and decomposition. The gas temperature and flow rate were not uniform in the boiler’s gas flue, and thus the evaporation rate of H_2_O_2_ in different locations was very different. If the injector position was not appropriate, H_2_O_2_ could not decompose quickly, and much of the H_2_O_2_ did not oxidize NO well, which led to the low oxidation efficiency of NO and low utilization of H_2_O_2_. The size of the H_2_O_2_ droplets ejected by different atomization methods was different. The smaller the droplet size, the larger the gas-liquid surface area under the same injection flow, which was beneficial to improve the evaporation rate of H_2_O_2_. Under a certain flue gas temperature, converting hydrogen peroxide solution to small droplets or thin liquid films would greatly improve the rate of evaporation and decomposition, thus ensuring high NO oxidation efficiency.

### Effect of gas temperature

**Fig 5:**
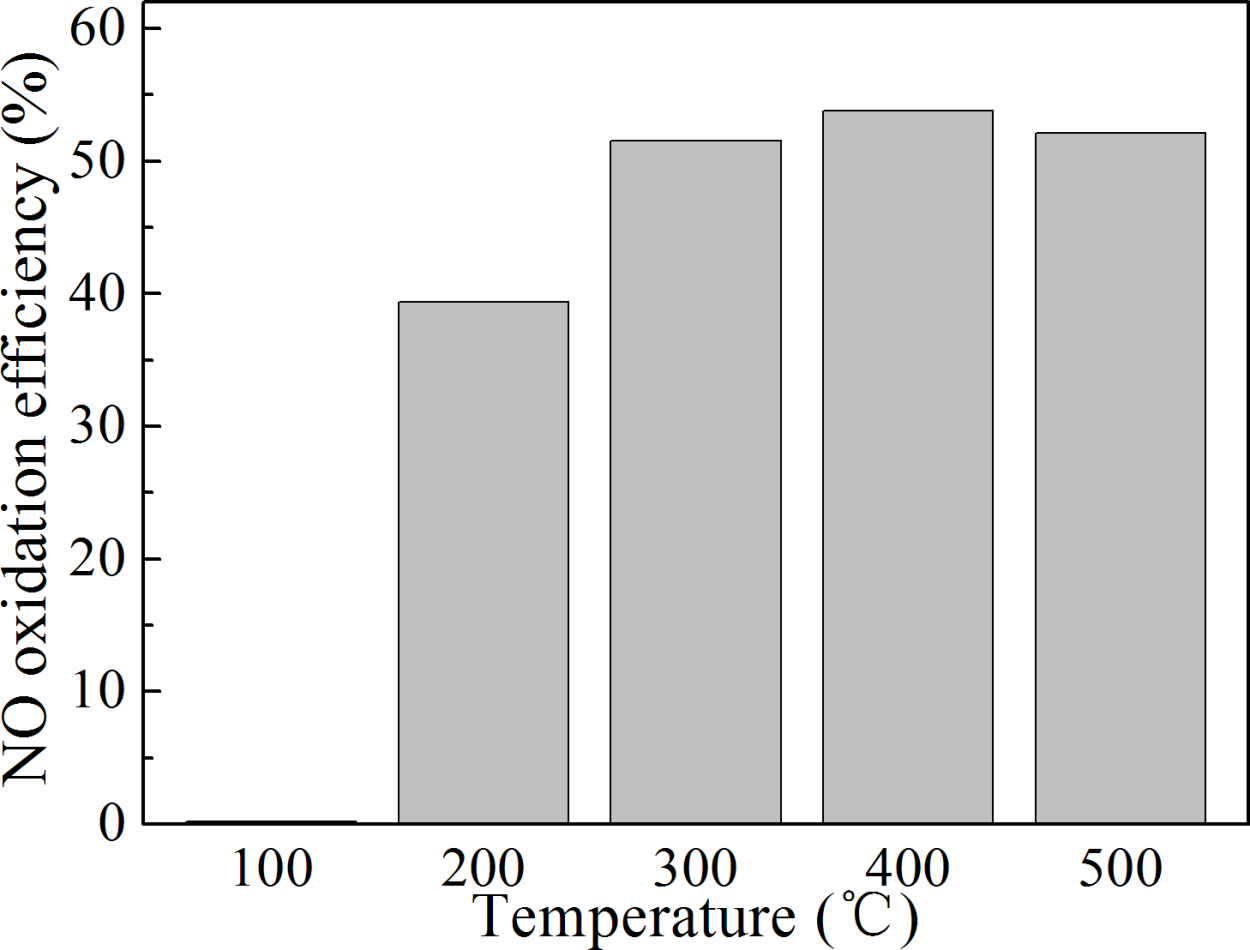
Influence of gas temperature on NO oxidation. The temperature in the reactor was between 100–500°C.

**Fig 6:**
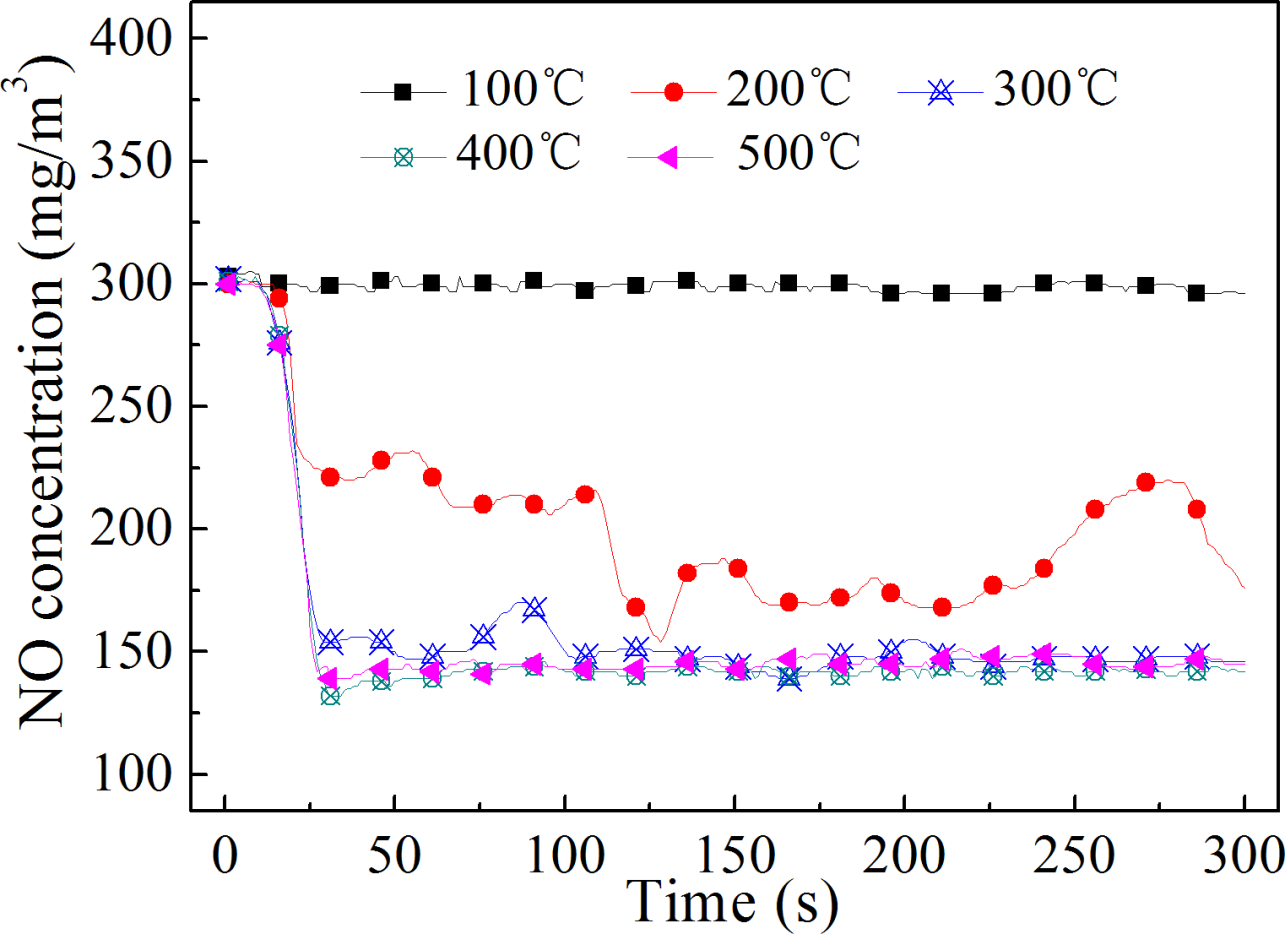
NO concentration at different temperatures. The temperature in the reactor was between 100–500°C.

Temperature affects not only the evaporation and the decomposition of the H_2_O_2_ solution but also the rate of the NO oxidation reaction. The effect of gas temperature on NO oxidation was studied in this research. The results are shown in Figs 5 and 6. It can be seen from Fig 5 that the NO oxidation efficiency rose with an increase in the gas temperature between 100–500°C. At 100°C, H_2_O_2_ did not oxidize NO gas well. This was because the boiling point of pure H_2_O_2_ is approximately 150.2°C at atmospheric pressure, and the boiling point of H_2_O_2_ solution is higher than 100°C.[21] Therefore, the H_2_O_2_ solution could not evaporate efficiently at this temperature. This result also indicated that H_2_O_2_ did not oxidize NO directly, and the oxidation capacity of H_2_O_2_ to NO was mainly due to the free radicals produced from its decomposition. Compared to that at 100°C, the NO oxidation efficiency rose significantly at 200°C. Because 200°C is higher than the evaporation temperature of 30% H_2_O_2_ solution, the H_2_O_2_ solution could evaporate and decompose in order to oxidize NO. However, because the gas temperature was not very high, the solution evaporation rate was not fast enough. Liquid droplets could be seen at the bottom of the oxidation reactor. This illustrated that only a portion of the injected H_2_O_2_ evaporated and decomposed. From Fig 6, it can be seen that the NO concentration at 200°C varied greatly, which is similar to the result of experiment 1 (Fig 4) in which H_2_O_2_ did not evaporate quickly.

In the temperature range of 300–500°C, the NO oxidation efficiency was above 50%, and it changed slowly with an increase in the gas temperature. The maximum oxidation efficiency was obtained at 400°C. It was difficult to see droplets dripping to the bottom of the reactor when the temperature was higher than 300°C. The NO concentration had only small fluctuations at 300°C. The NO concentration remained low during the oxidation process so that the average oxidation efficiency of NO was high at temperatures over 300°C.

In the literature, the NO oxidation rate was reported to be high over a narrow temperature range near500 °C, and it decreased sharply when the temperature deviated (no matter increased or decreased) from 500 °C.[6] There are three reasons which cause this result. Firstly, when the temperature was too low, the H_2_O_2_ evaporation rate was too slow. Secondly, the reaction rate of the reaction between ·OH and NO decreased with the increase in temperature.[22] If the temperature was too high, NO oxidation rate would decline. Thirdly, the wall destruction of the free radicals was accelerated when the gas temperature was high. This result is very unfavourable to the implementation of this technology. Because the gas temperature is uneven the flue gas flows. Even in the same cross section, the flue gas temperature is quite different. When H_2_O_2_ is injected into a flue gas, the NO oxidation efficiency will be low because of the uneven temperature field. It is important to ensure that the NO can be efficiently oxidized over a wide temperature range.

The results in this research were quite different from that in the literature. High NO oxidation efficiency could be obtained from 300 °C to 500 °C when the H_2_O_2_ solution evaporated and decomposed rapidly. The reason was that the evaporation rate of injected H_2_O_2_ was accelerated by converting it into very thin liquid films on the surface of the stainless-steel gas pipe. High NO oxidation efficiency was obtained at 300 °C when the H_2_O_2_ evaporation rate was fast. This result indicated that it was possible to oxidize NO gas efficiently by injecting H_2_O_2_ solution into the real flue gas. There may be two main ways to accelerate the evaporation of the H_2_O_2_ solution. First, the droplet diameter can be reduced using advanced liquid atomization technology, such as ultrasonic atomizers. Second, adequate solid surfaces with high surface energy can be placed near the H_2_O_2_ solution injectors, on which the H_2_O_2_ solution could spread into very thin liquid films. As to the same liquid, the larger the solid surface energy, the smaller the contact angle of the liquid on the surface, and the easier it is to spread the liquid into the film.

### Effect of the initial concentration of NO

**Fig 7:**
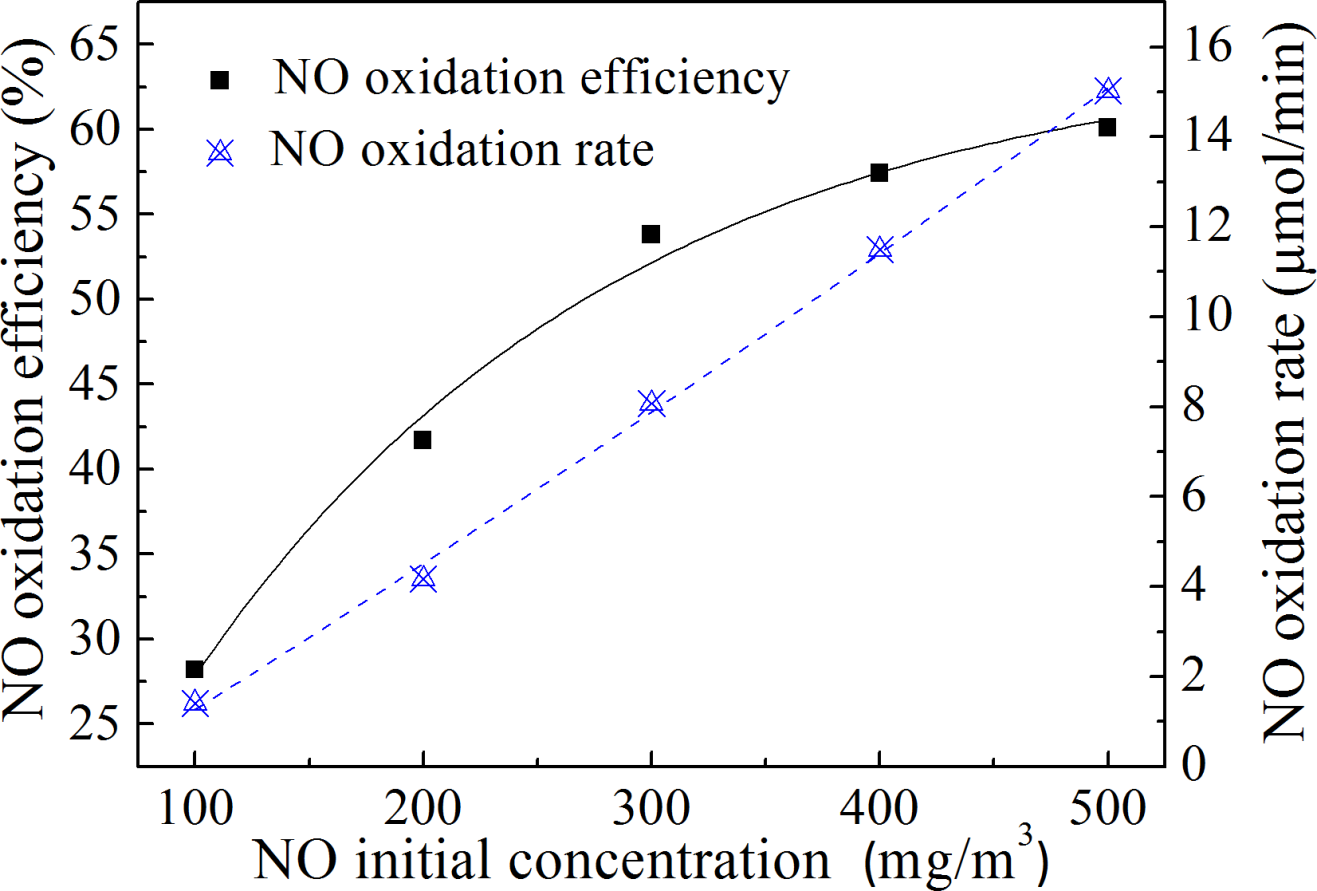
Influence of the initial NO concentration on NO oxidation. The temperature in the reactor was 400°C. The initial NO concentration ranged from 100–500 mg/m^3^.

There are significant differences in the NO*x* concentration in flue gas from different types of coal-fired boilers. When the boilers use low nitrogen combustion technology, the NO*x* concentration in the outlet flue gas is much lower. The influence of the initial NO concentration on the NO oxidation efficiency was studied in this experiment. As the concentration of the H_2_O_2_ solution did not change, the injection amount of the H_2_O_2_ solution was adjusted to maintain H_2_O_2_:NO = 10. The effect of the initial NO*x* concentration on the NO*x* oxidation efficiency is shown in Fig 7.

As shown in Fig 7, the NO oxidation efficiency increased monotonically with an increase in the initial NO concentration. The NO oxidation rate increased linearly with the increase in the initial NO concentration. This was because the higher the initial NO concentration, the faster the reaction rate between NO and ·OH radicals or HO_2_· radicals. As the gas flow rate was constant, the time that the N_2_+NO gas remained in the reactor did not change. The oxidation rate and oxidation efficiency of NO increased with an increase in the NO concentration over the same reaction time. It can be seen that this method was more effective for the oxidation of NO at high concentrations.

### Effect of H_2_O_2_ concentration

**Fig 8:**
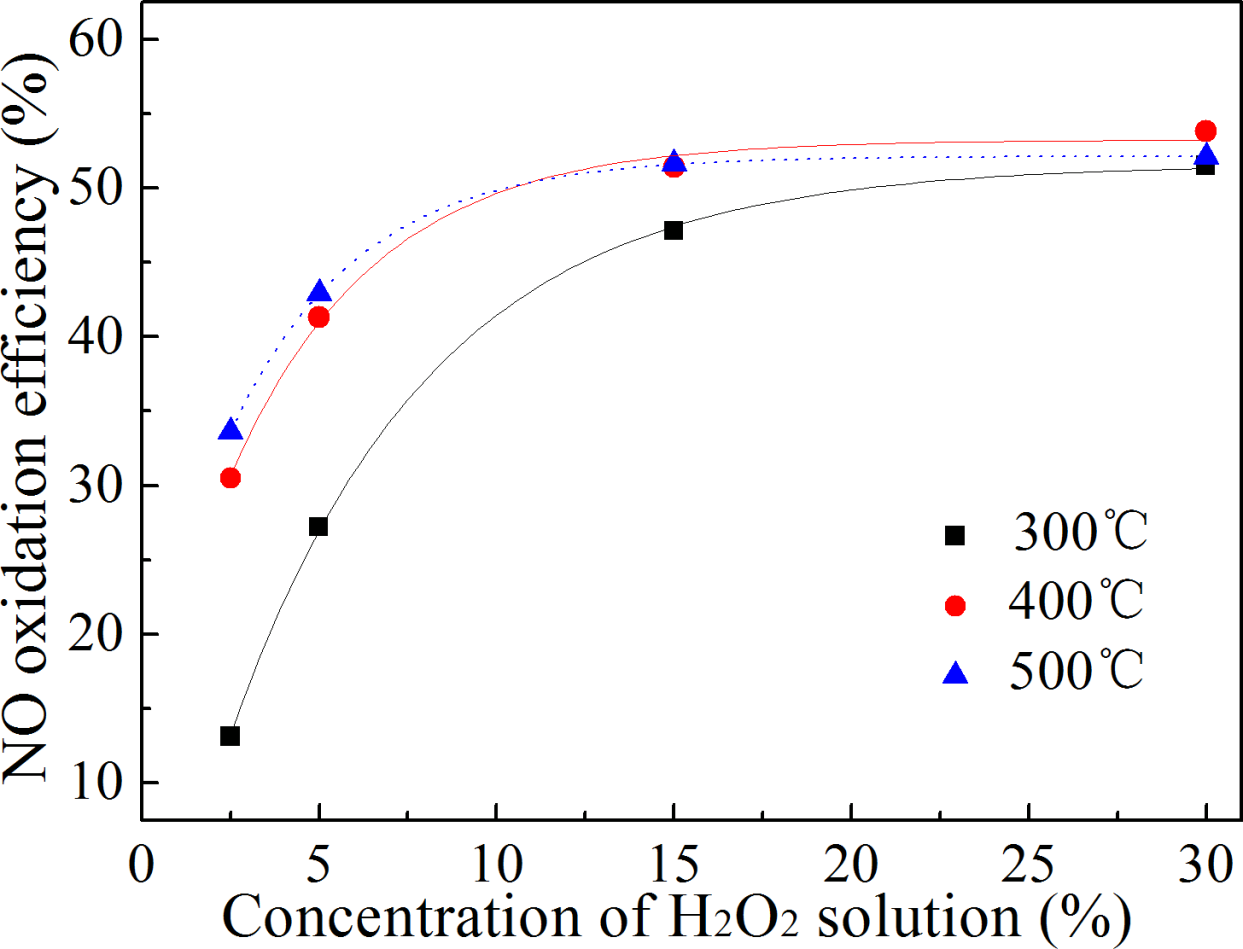
Effect of H_2_O_2_ concentration on NO oxidation efficiency. The concentration of the H_2_O_2_ solution was between 2.5%-30%. The temperature in the reactor was 300–500°C.

Under the same H_2_O_2_:NO molar ratio, the higher the H_2_O_2_ concentration, the less amount of H_2_O_2_ solution that needs to be injected. Water vapor absorbs less of the latent heat of vaporization, which has less impact on the gas temperature. When the injected H_2_O_2_ concentration is too low, more H_2_O_2_ solution is required to oxidize the same amount of NO. The flue gas temperature may decline significantly, and this will decrease the NO oxidation efficiency. Therefore, it is necessary to optimize NO oxidation using different concentrations of H_2_O_2_. The effect of the H_2_O_2_ solution concentration on the NO oxidation efficiency is shown in Fig 8.

Fig 8 shows that the higher the concentration of H_2_O_2_ solution under the same temperature conditions, the higher the NO oxidation efficiency. When the concentration was less than 15%, the NO oxidation efficiency increased with an increase in the H_2_O_2_ solution concentration. However, when the H_2_O_2_ concentration was higher than 15%, the NO oxidation efficiency increased only slightly. Because the H_2_O_2_:NO molar ratio remained constant, the volume of H_2_O_2_ solution injected into the gas could be decreased when using a high concentration of H_2_O_2_ solution. This would reduce the heat absorbed by the evaporation of water. When the concentration of the H_2_O_2_ solution was less than 15%, the large volume of H_2_O_2_ solution injected into the reactor greatly influenced the gas temperature, and the evaporation rate of H_2_O_2_ solution was greatly reduced, which decreased the oxidation efficiency of NO. It can also be seen from Fig 8 that the higher the gas temperature, the smaller effect of the H_2_O_2_ solution concentration on the NO oxidation efficiency. Considering the results of different gas temperature conditions, it is suggested that the H_2_O_2_ concentration is not lower than 15%.

In the previous studies, H_2_O_2_ solution with the concentration of 50% was used to oxidize NO.[6–8] The reason was that low concentrations of H_2_O_2_ seriously affected the solution evaporation rate, which decreased the NO oxidation efficiency. In this study, H_2_O_2_ evaporation rate was accelerated by spreading H_2_O_2_ solution into a thin film on the surface of the stainless-steel gas pipe. Under this condition, H_2_O_2_ concentration was decreased to 15%. The result illustrated that H_2_O_2_ solution with low concentration can be used to oxidize NO, as long as the evaporation rate is fast enough.

### Effect of H_2_O_2_:NO molar ratio

**Fig 9:**
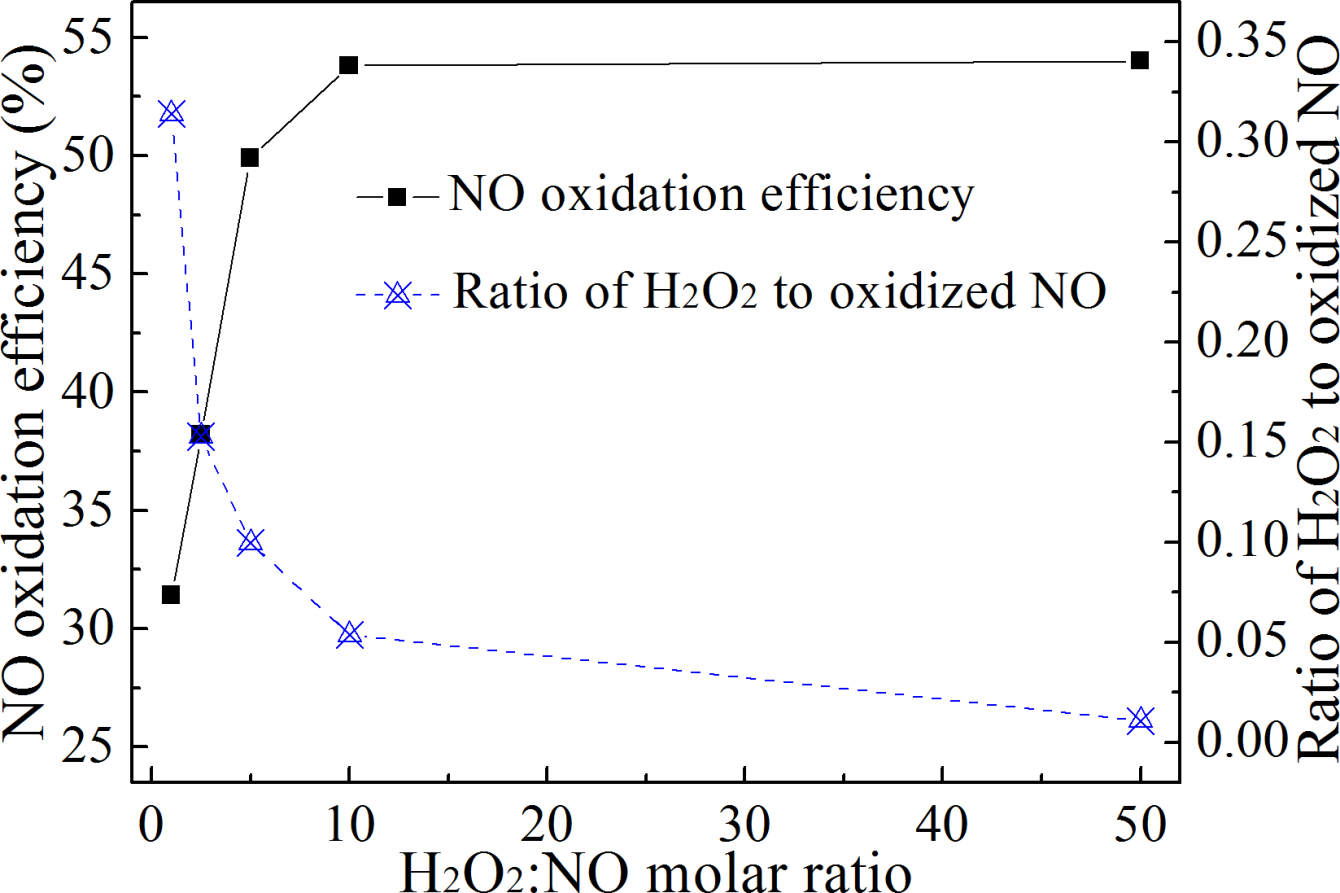
Influence of H_2_O_2_:NO molar ratio on NO oxidation. The H_2_O_2_:NO molar ratios were 1:1, 2.5:1, 5:1, 10:1 and 50:1. The temperature in the reactor was 500°C.

The H_2_O_2_:NO molar ratio directly determines whether the H_2_O_2_ injection method is economical compared to SCR technology. Haywood *et al*. proposed that at a molar ratio of 1.37:1, the method was an economically feasible alternative to the SCR method for NO*x* control.[4] If the NO in flue gas is pre-oxidized by injecting H_2_O_2_ and the oxidized N species (NO_2_ and HNO*x*) are removed along with SO_2_ by ammonia, the compound fertilizer of ammonium nitrate and ammonium sulfate will be produced. This compound fertilizer is expensive. Considering this by-product, the H_2_O_2_:NO molar ratio can be increased. However, the exact molar ratio needs to be recalculated. In this study, the NO oxidation efficiency was studied under different H_2_O_2_:NO mixture ratios. The results are shown in Fig 9.

Fig 9 shows that when H_2_O_2_:NO = 1, the oxidation efficiency of NO was 31.4%. The oxidation efficiency of NO increased with the an increase in the H_2_O_2_:NO molar ratio. After the H_2_O_2_:NO molar ratio reached 10, the NO oxidation efficiency did not increase with an increase in the molar ratio. Although the NO oxidation efficiency increased with the H_2_O_2_:NO molar ratio, the ratio of H_2_O_2_ to oxidized NO declined. This illustrated that the H_2_O_2_ utilization efficiency decreased with an increase in the molar ratio. Therefore, to reduce operation costs, it is crucial to obtain an appropriate NO oxidation efficiency by reducing the H_2_O_2_:NO molar ratio.

The result also proved the result in Fig 7, in which NO oxidation efficiency was only affected by NO concentration, but not H_2_O_2_ concentration. It can be seen from Fig 9 that when the molar ratio exceeded 10, the NO oxidation efficiency increased slowly. Under these conditions, the concentration of H_2_O_2_ increased with the increase of molar ratio, but it did not significantly promote NO oxidation efficiency. The results in Fig 7 were obtained at H_2_O_2_:NO = 10. When NO concentration increased from 300 mg/m^3^ to 500 mg/m^3^, H_2_O_2_ concentration also increased by 5/3 times to maintain H_2_O_2_:NO ratio.As a result, NO oxidation efficiency increased by 6.3% in Fig 7. However, if NO concentration maintained 300 mg/m^3^, H_2_O_2_ concentration still increased by 5/3 times, NO oxidation efficiency did not change significantly according to the result in Fig 9. Therefore, the increase of NO oxidation efficiency in Fig 7 must be the result of the effect of NO concentration.

### NO oxidation products

**Fig 10:**
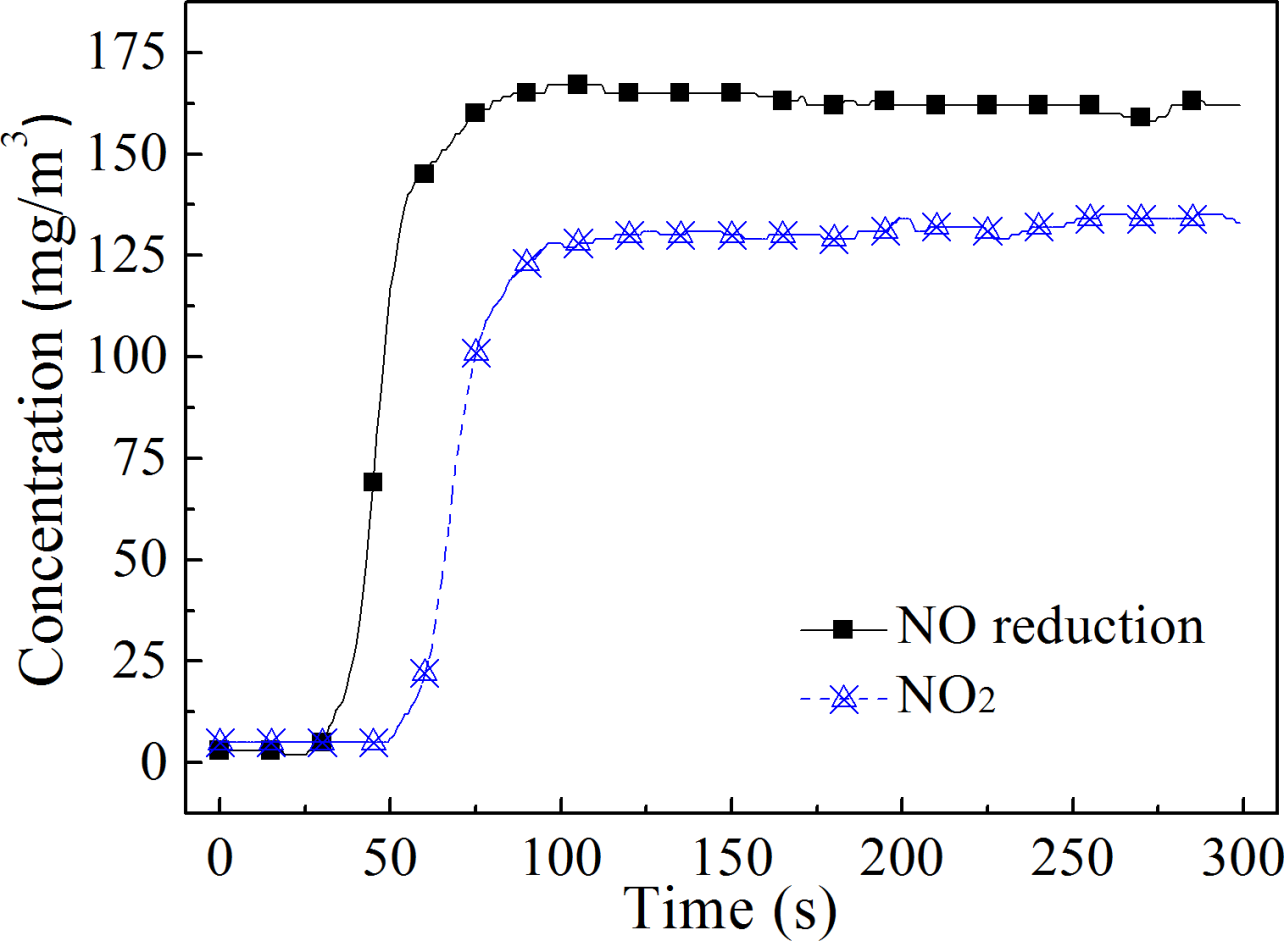
The change in NO_2_ concentration. The temperature in the reactor was 400°C.

The optimal oxidation ratio of NO was determined by the NO conversion products, as the dissolution characteristics of the various products are different. Limvoranusorn *et al*. [1] reported that the main products of NO oxidation by H_2_O_2_ thermal decomposition were NO_2_, HNO_2_ and HNO_3_. To obtain more soluble HNO*x*, a high NO oxidation ratio was pursued in their study. If the oxidation product is mainly NO_2_, a high NO oxidation ratio may not be conducive to the absorption of NO*x* because NO will be regenerated when NO_2_dissolves in water (reaction 6).[23,24] Some scholars have found that when the NO oxidation ratio was 50%-70%, the absorption rate of NO gas in alkaline solution was the highest.[20] Because equimolar amounts of NO and NO_2_aid in their dissolution (reaction 7),[23,24] it is very important to detect the products of NO oxidation by the H_2_O_2_ injection method to determine the reasonable oxidation ratio. The test results of the NO*x* concentration at the reactor outlet after injection of the H_2_O_2_ solution are shown in Fig 10.

$$3{\mathrm{NO}}_{2}+H_{2}O↔2{\mathrm{HNO}}_{3}+\mathrm{NO}$$ (6)

$$\mathrm{NO}+{\mathrm{NO}}_{2}+H_{2}O↔2{\mathrm{HNO}}_{2}$$ (7)

After the injection of H_2_O_2_, the NO concentration decreased significantly, and the NO_2_ concentration increased along with the reduction of NO. The NO concentration dropped from 300 mg/m^3^ to approximately 140 mg/m^3^, and the average NO reduction was 162.9 mg/m^3^. The NO_2_ concentration increased from 0 to approximately 130 mg/m^3^, and the average NO_2_ concentration was 131.4 mg/m^3^. The reduction in the NO concentration was only 31.5 mg/m^3^ higher than the NO_2_ concentration, which indicated that over 80% of the NO oxidation product was NO_2_. It can be deduced that reactions 1–5 were the primary reactions in the system. The reactions began with the decomposition of H_2_O_2_ (reaction 1), and then some of the ·OH and H_2_O_2_ reacted to generate HO_2_· radicals. Some of the ·OH radicals reacted with NO to form HNO_2_, and the generated HNO_2_ converted to NO_2_ quickly through reaction 3, followed by reaction 2. Reaction 8 was not the main reaction due to its slow reaction rate.[25]

$$H_{2}O_{2}\to \cdot \mathrm{OH}+\cdot \mathrm{OH}$$ (8)

NO+⋅OH→HONO (9)

$$\mathrm{HONO}+\cdot \mathrm{OH}\to {\mathrm{NO}}_{2}+H_{2}O$$ (10)

$$H_{2}O_{2}+\cdot \mathrm{OH}\to {\mathrm{HO}}_{2}\cdot +H_{2}O$$ (11)

$$\mathrm{NO}+{\mathrm{HO}}_{2}\cdot \to \cdot \mathrm{OH}+{\mathrm{NO}}_{2}$$ (12)

$${\mathrm{HO}}_{2}\cdot +\mathrm{NO}\to {\mathrm{HNO}}_{3}$$ (13)

### NO*x* absorption by NaOH solution

**Fig 11:**
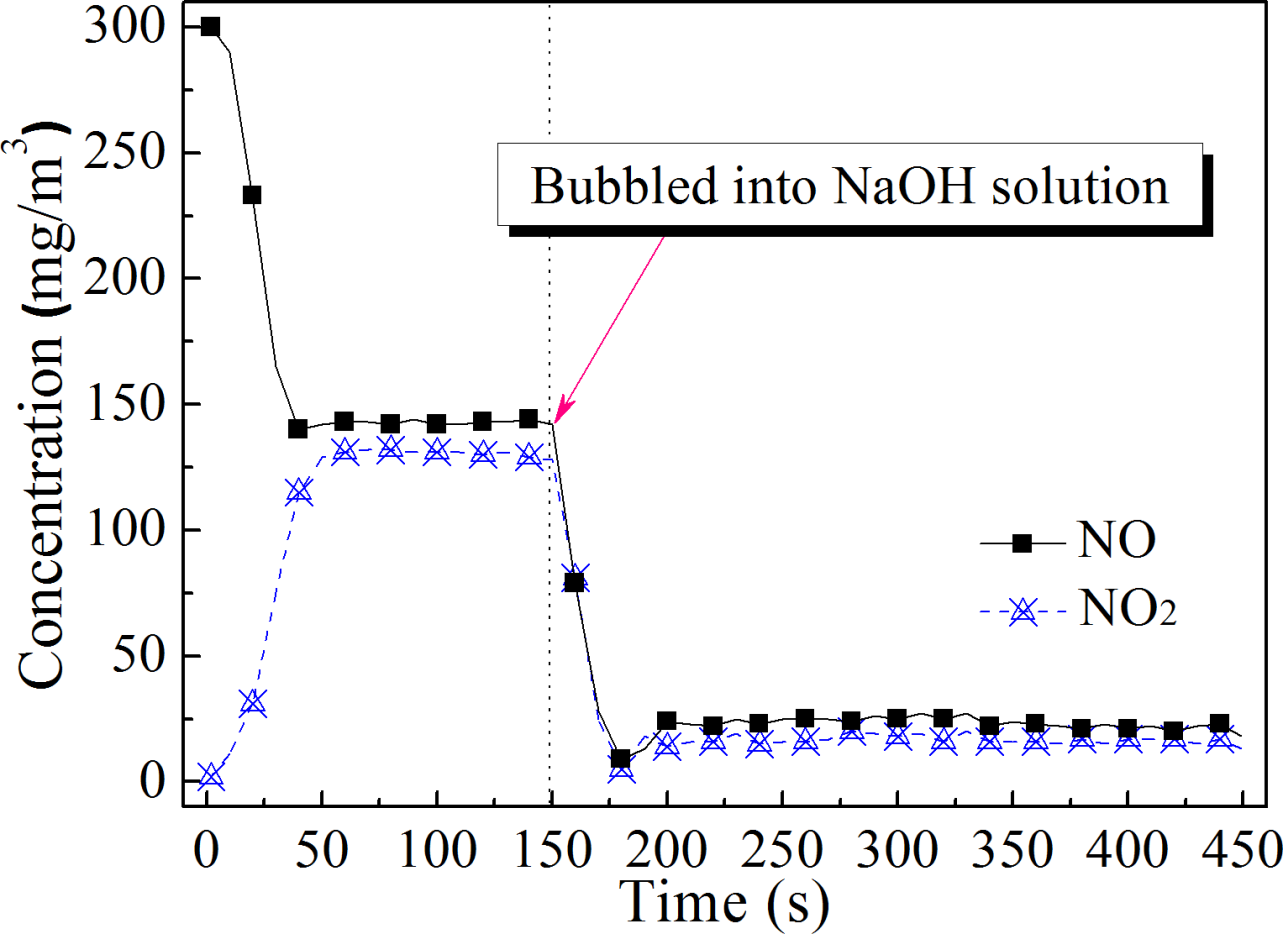
NO*x* removal by NaOH solution. The temperature in the reactor was 400°C. The concentration of the NaOH solution was 2 mol/L.

In this experiment, NaOH solution was used to absorb NO*x* from the gas in the oxidation reactor. The NaOH solution volume was 1.0 L, and the solution was placed in a bubbling reactor. The inner diameter of the bubbling reactor was 100 mm, and the volume was 1.09 L. The volume of gas in the bubbling reactor was small and thus had little effect on the concentration of NO*x* when the gas was bubbled into the reactor. After the hydrogen peroxide solution was injected into the oxidation reactor, the gas first entered the gas analyzer to record the NO*x* concentration change. When the NO and NO_2_ concentrations were stabilized, the gas from the oxidation reactor was directed into the NaOH solution. Then, the NO and NO_2_ concentrations were measured using the gas analyzer. The results of the NO*x* concentration measurements are shown in Fig 11.

In this experiment, the oxidation efficiency of NO was 53.8%, and the concentration of NO_2_ was slightly less than that of NO. It can be seen that the NaOH solution was very effective in absorbing NO*x* at this oxidation ratio. The average concentration of NO_2_ at the outlet of the bubbling reactor was 16.7 mg/m^3^, and the average concentration of NO was 23.3 mg/m^3^. The total NO*x* concentration was 40.0 mg/m^3^. The NO*x* removal efficiency was 86.7%. It can be seen that NO oxidation by H_2_O_2_ thermal decomposition is an ideal pre-oxidation technology, which when combined with alkali absorption, can achieve a high NO removal efficiency.

Due to the size of the oxidation reactor, the maximum oxidation efficiency of NO was approximately 54% at a NO concentration of 300 mg/m^3^, which was lower than that in the experiment of Collins and coworkers. However, they found that the NO oxidation efficiency in the pilot-scale experiment was much higher than that in the laboratory studies. If this technology is used to remove NO from real power plant flue gas, the H_2_O_2_:NO molar ratio would be significantly reduced, and the NO oxidation efficiency would be greatly enhanced. It is easy to obtain an NO oxidation efficiency of 50% at a very low H_2_O_2_:NO molar ratio. The required NO*x* emission concentration is less than 100 mg/m^3^ in China's new thermal power plant emission standards issued in 2011. This means that the NO*x* emission concentration from flue gases from most existing pulverized coal boilers would meet the new emission standards when using this method. The method may be an economic alternative to SCR technology.

This study confirmed that the oxidation efficiency did not need to be too high, and good removal could be obtained at an oxidation ratio of 50% when combined with alkali absorption. The temperature range for NO oxidation could be widened to 300–500 °C by the enhancement in the H_2_O_2_ evaporation rate. The injection position is conveniently behind the economizer, which makes the technology easy to implement. This method will be an economically feasible NO pre-oxidation technology for the simultaneous removal of SO_2_ and NO*x* using WFGD units.

## Discussion

In this study, H_2_O_2_:NO molar ratio was 10 in most experiments. Under these conditions, the generation rate of O_2_ was so low that it was difficult to detect. Only when H_2_O_2_:NO molar ratio was high (such as 50), a small amount of O_2_ (concentration below 0.3%) can be detected. There are two main reasons for the low O_2_ concentration. Firstly, the injection amount of H_2_O_2_ was relatively small, resulting in low O_2_ concentration. The initial concentration of NO was 300 mg/m^3^, and the total gas flow rate was 1.5 L/min. It can be calculated that the flow rate of NO was only 10^−5^ mol/min. When H_2_O_2_:NO = 50, the injection rate of H_2_O_2_ was 5×10^−4^ mol/min. The maximum generation rate of O_2_ was 2.5×10^−4^ mol/min (2H_2_O_2_→O_2_). The concentration of O_2_ in the flow was 2.5×10^−4^×22.4/1.5×100% = 0.37%, which was very low. Secondly, not all free radicals generated from the decomposition of H_2_O_2_ were involved in O_2_ generation reactions. Many free radicals reacted with NO, H_2_O_2_ or the mid products (reaction 2–5), which decreased the yield of O_2_.

In addition, reaction 9 is a third order reaction. The reaction rate constant is only 3.30×10^−39^×e^-4.41/RT^ cm^6^/molecule^2^ s, which is much lower than reaction 2 (3.30×10^−11^ cm^3^/molecule s).[22]

$$NO+NO+O_{2}\to NO_{2}+NO_{2}\left(9\right)$$

We have tried the NO oxidation by 0.3% O_2_ at 500 °C, however, the concentration of NO did not changed. Based on the above analysis, we believe that the oxidation of NO by O_2_ is negligible.

In addition, when the NO initial concentration was 300mg/m^3^, the maximum NO oxidation efficiency was less than 55%. We found that the maximum oxidation efficiency was limited by the size of the reactor. We have tried two reactors with different size. In this study, the larger reactor was used. The length of the larger reactor was 680 mm and the inner diameter was 50 mm. The flow rate was 1.5 L/min, so the residence time was 54 s. The smaller reactor was 500 mm long and 38 mm in diameter. The flow rate was 0.65 L/min, and the residence time was 52 s. Due to the high rate of the reaction between NO and ·OH, the residence time was adequate. The other parameters such as temperature, initial concentration of NO, H_2_O_2_:NO were the same. Though the residence time in two reactors was almost the same, the maximum NO oxidation efficiency had a great difference. The maximum NO oxidation efficiency in the larger reactor was near 55%, but the maximum NO oxidation efficiency in the smaller reactor was only 43%. The higher oxidation efficiency in the larger reactor was not due to the higher injection amount of H_2_O_2_. Because the concentrations of H_2_O_2_ in the gases were the same, we believe that the free radicals have a wall destruction effect. The wall in the large reactor is far from the reaction zone, which weakened the useless destruction of the free radicals.

## Conclusions

The general conditions of NO oxidation by H_2_O_2_ thermal decomposition were investigated in the drop-tube furnace NO oxidization experimental system. The conclusions are as follows:

1. Sufficient evaporation of the H_2_O_2_ solution was a prerequisite to achieve the high oxidation efficiency of NO. The NO oxidation efficiency could be obtained over a wide temperature range when the H_2_O_2_ solution evaporated and decomposed rapidly. The NO oxidation efficiency was above 50% in the temperature range of 300–500°C in this research.
2. The NO oxidation efficiency and the NO oxidation rate increased with an increase in theinitial NO concentration. This method was more effective for the oxidation of NO at high concentrations.
3. High temperatures decreased the influence of the H_2_O_2_ concentration on the NO oxidation efficiency. The higher the concentration of H_2_O_2_ solution under the same temperature conditions, the higher the NO oxidation efficiency. Considering the results of different gas temperature conditions, it is suggested that the H_2_O_2_ concentration is not lower than 15%.
4. The oxidation efficiency of NO increased with an increase in the H_2_O_2_:NO molar ratio, but the ratio of H_2_O_2_ to oxidized NO decreased. Oxidizing NO at an appropriate ratio with a low H_2_O_2_:NO molar ratio could reduce the cost of NO oxidation.
5. It was proven that the NO oxidation product was mainly NO_2_ and that the oxidation ratio of NO did not need to be very high. An 86.7% NO removal efficiency was obtained at an oxidation ratio of 53.8% when combined with NaOH solution absorption.
